# Correction to “In‐Hospital Nursing Care Intervention Increasing the Effect of Vacuum Sealing Drainage on Wound Healing: A Meta‐Analysis”

**DOI:** 10.1111/iwj.70834

**Published:** 2026-01-25

L. Yu, Y. Wang, D. Ma, et al., “In‐Hospital Nursing Care Intervention Increasing the Effect of Vacuum Sealing Drainage on Wound Healing: A Meta‐Analysis,” *International Wound Journal* 20, no. 8 (2023): 3371–3379, https://doi.org/10.1111/iwj.14169.

In the Materials and Methods section, the sentence “Then, the rest of 92 studies were checked, and 47 studies were excluded for their irrelevancy to our study objective” was incorrect. It should have been: “Then, the rest of 92 studies were checked, and 69 studies were excluded for their irrelevancy to our study objective.”

We apologise for this error.

